# Loss of coordination between basic cellular processes in human aging

**DOI:** 10.1038/s43587-024-00696-y

**Published:** 2024-09-03

**Authors:** Ana Carolina Leote, Francisco Lopes, Andreas Beyer

**Affiliations:** 1 Cologne Excellence Cluster on Cellular Stress Responses in Age-Associated Diseases (CECAD), University of Cologne, Cologne, Germany; 2 Department II of Internal Medicine, University of Cologne, Faculty of Medicine and University Hospital Cologne, Cologne, Germany; 3 Institute for Genetics, Faculty of Mathematics and Natural Sciences, University of Cologne, Cologne, Germany; 4 Center for Molecular Medicine Cologne (CMMC), University of Cologne, Faculty of Medicine and University Hospital Cologne, Cologne, Germany

**Keywords:** Transcriptomics, Regulatory networks, Gene regulatory networks, Ageing

## Abstract

Age-related loss of gene expression coordination has been reported for distinct cell types and may lead to impaired cellular function. Here we propose a method for quantifying age-related changes in transcriptional regulatory relationships between genes, based on a model learned from external data. We used this method to uncover age-related trends in gene–gene relationships across eight human tissues, which demonstrates that reduced co-expression may also result from coordinated transcriptional responses. Our analyses reveal similar numbers of strengthening and weakening gene–gene relationships with age, impacting both tissue-specific (for example, coagulation in blood) and ubiquitous biological functions. Regulatory relationships becoming weaker with age were established mostly between genes operating in distinct cellular processes. As opposed to that, regulatory relationships becoming stronger with age were established both within and between different cellular functions. Our work reveals that, although most transcriptional regulatory gene–gene relationships are maintained during aging, those with declining regulatory coupling result mostly from a loss of coordination between distinct cellular processes.

## Abstract (web summary)

Age-related loss of gene expression coordination may contribute to impaired cellular function. In this study, the authors analyzed age-related trends in gene–gene relationships. They found that expression control often remains intact among genes involved in shared cellular functions while the coordination between functions tends to decline.

## Main

Investigation of transcriptomic signatures of aging has uncovered alterations in several processes, either as possible causes of cellular damage or as adaptive responses to age-related functional decline^1^. Age-associated changes of basic cellular processes, such as the accumulation of DNA damage, loss of proteostasis, deregulated nutrient sensing, impaired mitochondrial function, accumulation of senescent cells, exhaustion of stem cell niches and altered intercellular communication, all result in transcriptomic changes. Although age-related changes within each of these processes have become better understood, many of the unanswered questions now revolve around the impact of aging on the regulation of each of these processes individually and especially the coordination with other processes.

One of the standing questions in the field is how universal is age-related gene expression deregulation, if it takes place at all^2^. Early work reported an age-related increase in the variability (noise) of expression levels of individual genes across cells in some cell types but not others^3,4^. Further work making use of single-cell RNA sequencing (scRNA-seq) data has led to the conclusion that increased cell-to-cell variation is not universally observed across all genes and cell types^5–7^.

Importantly, increasing expression heterogeneity does not necessarily result from a loss of transcriptional coordination. Instead, variability may also arise due to the coordinated response of cells to variable external stimuli. It remains an open question to what extent coordination of gene expression becomes impaired with age. Because genes operate in functional modules^8,9^, such as protein complexes or pathways, expression coordination within and between gene modules is required to orchestrate a functional cellular response. For instance, the impact of aging on the coordination of T cell activation in response to stimuli has been addressed using scRNA-seq data to show that T cell activation is impaired (weaker and more variable) in old mice compared to young mice^10^. Genome-wide analysis of transcription factor (TF) activity in scRNA-seq data from immune cells also revealed age-related alterations in regulatory factor activity and macrophage dedifferentiation^11^. Given the complexity of regulatory interactions between cellular processes, addressing the impact of aging on within-module and between-module coordination requires a systems-level approach. Levy et al.^12^ developed a metric of global transcriptomic coordination that decreased with age in different organisms and cell types. Although this metric quantifies transcriptomic coordination at a large scale, it does not provide a quantification of coordination loss between specific transcriptional processes or gene pairs. To answer these questions, the sparsity of scRNA-seq data and the still comparably small amount of data collected for different ages and tissues poses a challenge, especially for a systematic cross-tissue analysis. Bulk RNA-seq data have been used successfully in the past to find a tendency for a global decrease in pairwise gene correlations in old mice, impacting ribosome biogenesis, transcriptional regulation and mitochondrial functions, along with an increase in correlations between DNA damage genes^13^. However, a comprehensive, cross-tissue analysis of the impact of aging on the coordination within and crosstalk between individual gene modules is still lacking.

Here we describe an approach for the identification of alterations in gene–gene relationships with age in humans. First, we report a regulatory model, derived from more than 1,000 expression datasets, that is capable of capturing tissue-specific and global (cross-tissue) gene–gene relationship changes with age. Motivated by this wide applicability, we used RNA-seq data of 30 human tissues at different ages to investigate age-related changes in gene–gene relationships. Our analysis revealed tissue-specific and generic (across tissues) changes in gene expression coordination between individual gene pairs and entire modules. Notably, we found similar numbers of genes with increasing and decreasing expression coordination with age. We observed the most widespread changes in expression coordination among genes involved in mitochondrial respiration and cell cycle regulation, and we provide examples of age-related regulation changes in tissue-specific functions as well as in the crosstalk between different cellular functions.

## Results

### Capture of gene–gene relationships from transcriptome data

**Fig. 1:**
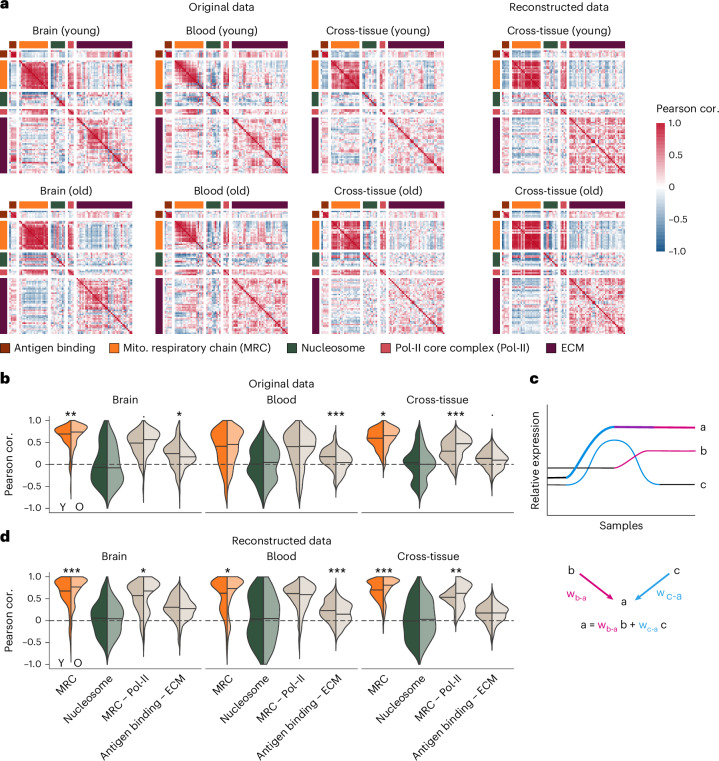
Representative gene–gene relationship changes with age, captured by pairwise correlation and a GRN-based approach. **a**, Heatmap of pairwise Pearson correlation coefficients for selected cellular functions (modules) and tissues. Correlations were computed based on the original expression data (Original) or expression data reconstructed based on model predictions from **c** (Reconstructed), in young (20–29 years) or old (60–69 years) samples from Brain, Blood or pooled GTEx tissues (Cross-tissue). **b**,**d**, Quantification of within-module (colored) and between-module (beige) correlations for selected modules, in young (opaque) and old (transparent) samples. Correlations were computed based on the original expression data (**b**) or expression data reconstructed based on model predictions from **c**. **d**, Between-module correlations are presented for selected module pairs. A two-sided Mann–Whitney test was used to identify modules with age-related correlation changes: MRC (*P* = 0.0034 in the Original Brain data, *P* = 0.000094 in the Reconstructed Brain data, *P* = 0.018 in the Reconstructed Blood data, *P* = 0.047 in the Original Cross-tissue data, *P* = 0.00033 in the Reconstructed Cross-tissue data); MRC – Pol-II (*P* = 0.055 in the Original Brain data, *P* = 0.030 in the Reconstructed Brain data, *P* = 0.00083 in the Original Cross-tissue data, *P* = 0.0068 in the Reconstructed Cross-tissue data); Antigen binding – ECM (*P* = 0.023 in the Original Brain data, *P* = 4.7 × 10^−10^ in the Original Blood data, *P* = 0.00028 in the Reconstructed Blood data, *P* = 0.072 in the Original Cross-tissue data). **c**, Methodological approach used to capture gene–gene relationships in our regulatory model. Through a combination of regularized linear regression (LASSO) and stability selection, we identified stable predictors for each gene in the transcriptome—that is, genes whose expression pattern across the training data (cancer cell line transcriptomic data) is informative of the expression pattern of the target gene. A linear model was then fit to explain the expression pattern of the target gene (a) based on the pattern of the stable predictors (b and c). The weights of this linear model can then be used in other datasets to reconstruct the expression pattern of the target gene a based on the expression pattern of the stable predictors b and c observed in those datasets. Although the scheme shows two predictors, b and c, for target gene a, the number of predictor genes is not limited; rather, the optimal number of predictors is determined individually for each target gene (Methods). For illustrative purposes, gene sets were restricted to the following functions: respiratory chain complex members I–IV (MRC, GO:0045271, GO:0005749, GO:0005750 and GO:0005751, orange), components of collagen-containing ECM (GO:0062023, purple), Pol-II core complex members (GO:0005665, pink), nucleosome members (GO:0000786, dark green) and peptide antigen binding partners (GO:0042605, brown). ***P < 0.001; **P < 0.01; *P < 0.05; ^.^*P* < 0.1. cor., correlation; Mito., mitochondrial.

To investigate age-related changes in gene co-expression, we made use of RNA-seq data collected postmortem by the Genotype-Tissue Expression (GTEx) consortium^14^ from 30 different human tissues, spanning 948 donors aged between 20 years and 79 years. We first aimed to illustrate age-related changes in gene expression coordination within and between modules. For this, we hand-picked five sets of genes, constructed based on Gene Ontology (GO) term membership. This selection includes both cell-type-specific (for example, antigen binding) and ubiquitous (for example, mitochondrial respiratory chain) gene sets as well as both broad regulatory groups (for example, extracellular matrix (ECM) components) and protein complexes (for example, polymerase II (Pol-II) core complex). We computed all pairwise Pearson correlations in samples collected from either young (20–29 years) or old (60–69 years) donors. We focused our analyses on two tissues with differences in cellular composition and function—Brain and Blood—and on data from all tissues pooled together (Methods). We observed strong within-module correlations across ages and tissues (Fig. 1a, original data, diagonal blocks, and Fig. 1b, colored), in line with a modular organization of gene expression. Additionally, we observed between-module correlations (Fig. 1a, original data, off-diagonal blocks, and Fig. 1b, beige), particularly between genes encoding for members of the mitochondrial respiratory chain and the RNA Pol-II core complex (Fig. 1a,b), representative of regulatory crosstalk between functional modules. Both within-module and between-module correlations differed between tissues and age groups. Notably, we observed age-related differences in gene co-expression both specific to individual tissues (Fig. 1a,b, antigen binding – ECM in blood) and shared across tissues (Fig. 1a,b, mitochondrial respiratory chain in the cross-tissue analysis). These examples confirm the notion that gene co-expression relationships change with age. However, reduced co-expression between individual gene pairs does not necessarily result from reduced expression coordination. Instead, it may also result from adaptive changes requiring a different regulation of the respective genes.

Therefore, we next aimed to systematically map out robust regulatory expression relationships between genes that better reflect functional requirements for coordinated expression than simple pairwise co-expression. To capture robust regulatory relationships that explain expression coordination across different tissues, we inferred gene–gene relationships using transcriptomic data collected from 1,443 cancer cell lines^15,16^. The benefit of using in vitro gene expression data for model training, as opposed to using tissue data, is the fact that such data do not result from a mix of different cell types and are not affected by changes in cell type composition. As opposed to single-cell data, bulk in vitro data provide greater coverage and sensitivity. To derive gene expression relationships from large transcriptomic datasets, we devised a procedure that selects a small set of genes whose expression patterns are, as a linear combination, most predictive for the expression of a given target gene (as in refs. ^11,17,18^). The goal of this model is not to predict the global average expression level of a gene but the deviation from this global average in a given individual—that is, predicting inter-individual variation in gene expression. The network resulting from this procedure is much sparser than computing all pairwise correlations between all genes: only a very small fraction (0.04%) of gene pairs has non-zero relationships (ignoring directionality). These gene–gene relationships do not exclusively represent causal relationships between regulators and their targets. Instead, they represent gene pairs that are robustly co-expressed across a wide range of human tissues and cell types^16^.

**Extended Data Fig. 1:**
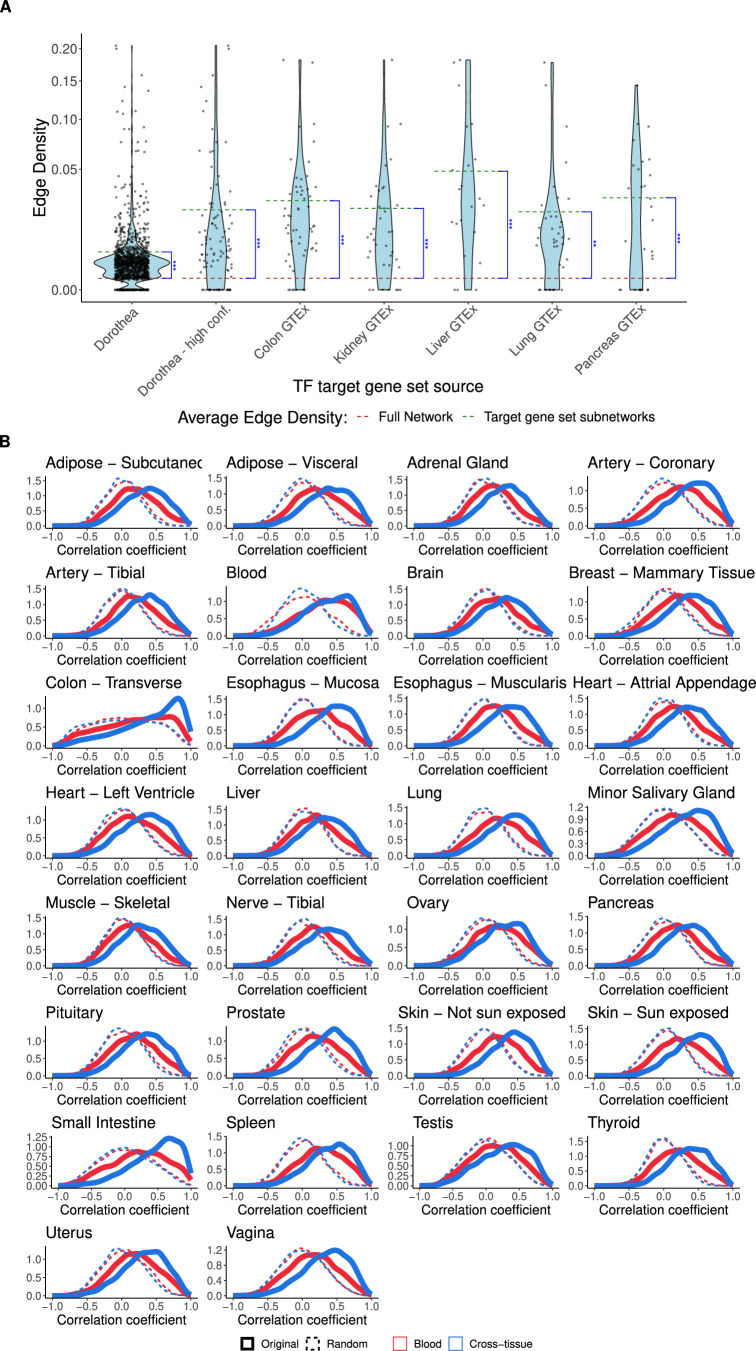
Network quality control. **a) Distribution of edge densities for transcription factor (TF) target gene sets**. Tissue-unspecific TF target gene sets were obtained from Dorothea^19^ (Dorothea, Dorothea - high conf.). Tissue-specific TF target sets were obtained from^18^ and are based on GTEx data (Colon GTEx, Kidney GTEx, Liver GTEx, Lung GTEx and Pancreas GTEx). Edge density for a gene set was defined as the fraction of existing edges among members of the gene set divided by the maximum possible number of edges (Methods). Each dot represents the edge density of one TF target gene set. A random subset of genes from the network is expected to have on average the same edge density as the full network. Therefore, the average edge density across TF target gene sets (green dashed line) was compared to the edge density of the full network (red dashed line) using one-sample one-sided t-tests and found to be significant in all cases (Dorothea p-value = 1.6 × 10^−29^, Dorothea – high conf. p-value = 5.6 × 10^−7^, Colon GTEx p-value = 5.6 x10^−7^, Kidney GTEx p-value = 1.9 × 10^−4^, Liver GTEx p-value = 3.0 × 10^−4^, Lung GTEx p-value = 1.0 × 10^−3^, Pancreas GTEx p-value = 8.1 × 10^−5^). ‘***’: p-value < 0.001; ‘**’: p-value < 0.01. **b) Performance of cross-tissue (blue) and blood-specific (red) models on GTEx tissues**. The expression of each gene was predicted in a given GTEx sample using the measured expression values of all detected predictors in each model (Methods). Subsequently, observed and predicted expression values for each gene were correlated across all samples from each tissue (Spearman correlation). The cross-tissue model matches the blood-specific model in Blood and performs better across all remaining tissues.

Next, we assessed the capacity of our model to capture known direct regulatory relationships between regulatory TFs and their target genes. We collected gene sets corresponding to TF targets from the Dorothea database^19^ and tissue-specific TF–target relationships inferred from the GTEx data^18^. For each of those sets, we computed edge connectivity in our network (Methods) and observed that the connectivity of genes regulated by the same TF was consistently and significantly higher than the background network connectivity (Extended Data Fig. 1a). This was true for both the cross-tissue (Dorothea sets) and tissue-specific (GTEx tissue sets) TF target gene sets. This result supports the notion that the connectivity patterns in our network capture generic and tissue-specific regulatory programs.

Using the gene–gene relationships captured by our regulatory model, we reconstructed the expression pattern of each gene across samples of the same tissue (Fig. 1c and Methods). Thus, the reconstructed data consist of the expected (predicted) pattern for each gene given the expression pattern observed for its regulatory neighbors (that is, directly connected genes in the network, corresponding to the most strongly co-regulated gene pairs). First, we observed that tissue-specific differences in the gene–gene correlation patterns observed in the original data (Fig. 1a) were largely preserved in the reconstructed data (Extended Data Fig. 3)— for instance, the weaker correlation between a subset of the members of the mitochondrial respiratory chain in Blood but not Brain. This was true not only for within-module correlations but also for correlations between different modules, as is the case between genes encoding for peptide antigen binding partners and ECM components. This observation confirms that our network captures regulatory neighborhoods that are conserved across cell types and tissues and, at the same time, enables prediction of tissue-specific expression levels. Second, we observed that age-related co-expression changes observed in the original data (Fig. 1a,b) were also captured in the reconstructed data (Fig. 1d and Extended Data Fig. 3). This observation is important because it supports the notion that co-expression changes (especially co-expression reduction) do not necessarily result from ‘sloppy regulation’ but may reflect regulated, physiologically plausible cellular responses.

The results shown in Fig. 1 are limited to a small subset of hand-picked genes and tissues to illustrate aspects of age-related changes in gene expression coordination, but they were not intended to replace a systematic, transcriptome-wide analysis. Therefore, we next tested the predictive power of our network model across all genes and tissues present in the GTEx dataset. Inter-individual differences in gene expression were predicted better than random guessing for the vast majority of genes and tissues (Extended Data Fig. 1b, blue distributions, and ref. ^16^). Thus, the network model captures gene–gene relationships that are conserved across a large diversity of cellular states. However, we note that the capacity of our model to correctly capture relevant regulatory neighborhoods is dependent on the gene and dataset at hand^16^. To gain a better understanding of the limitations of our model, we identified genes whose observed expression profiles differ strongly from the predicted based on their regulatory neighborhood (Methods). Our analyses revealed that most of these poorly predicted genes fell into one of two groups. The first group consisted of lowly expressed genes with few regulatory neighbors, which themselves were also lowly expressed (Extended Data Fig. 4). Thus, these genes correspond to network regions that were essentially inactive in the respective tissue or cell type, and, therefore, the observed expression variation reflected mostly background noise. The second group corresponded to highly expressed genes with extremely low variance and ubiquitous expression across tissues (Extended Data Fig. 4), suggesting that these are housekeeping genes with essentially constant expression. Because our model predicts expression variation across samples (that is, relative differences in expression between different samples), a constant expression results in virtually no relative differences between samples and is, thus, hard to model. Additionally, we functionally characterized genes based on the agreement/disagreement between observed and predicted expression levels across tissues (Methods). We consistently found genes involved in general cellular functions, such as RNA binding, translation and oxidative phosphorylation, to be well predicted across tissues, whereas genes encoding for signaling receptors were poorly predicted (Extended Data Fig. 4f). In subsequent analyses, we excluded genes that were not predictable by our model in a given tissue—that is, we used our network model only in a gene–tissue context where it is actually applicable.

**Extended Data Fig. 2:**
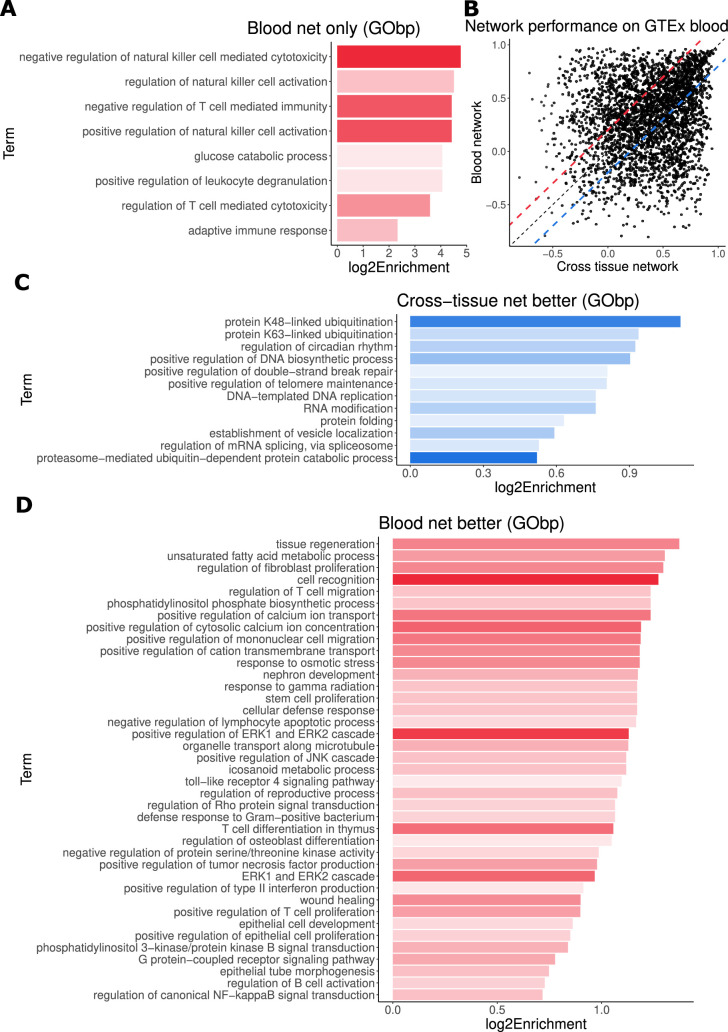
Performance comparison between cross-tissue and blood-specific models. **a)** GO biological process terms enriched in genes uniquely predicted by the blood-specific model. **b)** Scatterplot of performance (Spearman correlation) of both models in the GTEx Blood RNA-seq data. Colored dashed lines indicate a ± 0.2 correlation window around the diagonal (dashed black line), used to select genes better predicted by each of the models. **c)** GO biological process terms enriched in genes better predicted by the cross-tissue model. **d)** GO biological process terms enriched in genes better predicted by the blood-specific model.

**Extended Data Fig. 3:**
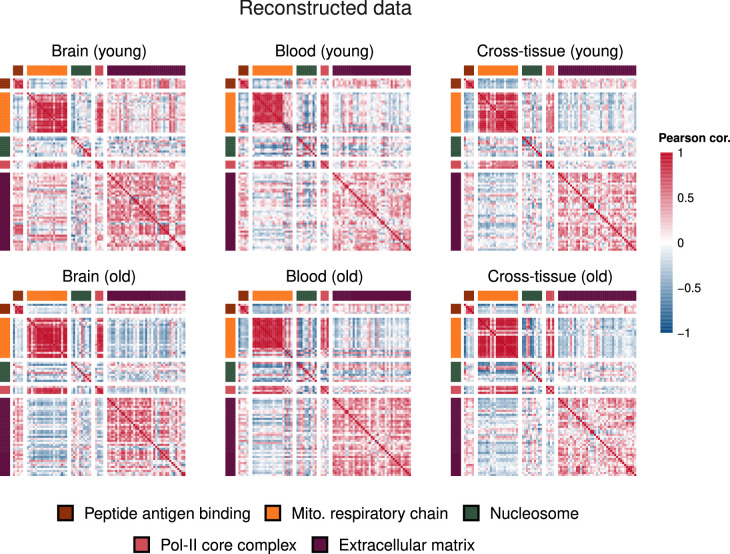
Representative gene-gene relationship changes with age, captured by a gene regulatory network (GRN)-based approach. Heatmap of pairwise Pearson correlation coefficients for selected cellular functions (modules) and tissues. Correlations were computed based on the expression data reconstructed based on model predictions (Fig. 1c, Methods), in young (20–29y) or old (60–69y) samples from Brain, Blood, or pooled GTEx tissues (Cross-tissue). For illustrative purposes, gene sets were restricted to the following functions: respiratory chain complex members I to IV (GO:0045271, GO:0005749, GO:0005750, GO:0005751, orange), components of collagen-containing extracellular matrix (GO:0062023, purple), polymerase II core complex members (GO:0005665, pink), nucleosome members (GO:0000786, dark green) and peptide antigen binding partners (GO:0042605, brown).

Next, we asked how much the model predictions would improve if tissue-specific networks were available. We, therefore, retrieved publicly available scRNA-seq data collected from peripheral blood mononuclear cells (PBMCs) from healthy donors^20^ and trained a blood-specific model using the same strategy as before. Compared to the cross-tissue (cancer cell line–based) network, the blood network performed worse on all GTEx tissues, except for GTEx blood, where both networks performed equally well. (Methods and Extended Data Fig. 1b). Further comparison of these two networks revealed a much higher coverage of the cross-tissue network (16,624 predictable genes) compared to the blood-specific network (4,141 predictable genes). The expression pattern of genes involved in blood-specific regulatory functions was better predicted by the blood-specific network, whereas the cross-tissue network performed better for genes involved in basic cellular functions (Extended Data Fig. 2). We concluded from this analysis that most gene–gene relationships captured by the cross-tissue network are conserved across cell types, and the potential benefit of having tissue-specific or cell-type-specific networks is partially offset by the larger training dataset available for the cross-tissue network.

### Age-related changes in gene expression coordination

Next, we systematically investigated changes in gene regulatory programs across the entire transcriptome and across human tissues. Here, we aimed to separate those changes in regulatory programs from changes in gene expression levels. As described above, our network model consists of gene–gene relationships that are invariant across a large diversity of cellular states. Thus, our notion was that changes in expression coordination would modify the ability of our network model to correctly predict the expression of a gene. Whenever the regulation of a gene ‘aligns’ with the model structure, it will be predictable, whereas regulatory inputs deviating from the model structure would reduce its predictability.

**Fig. 2:**
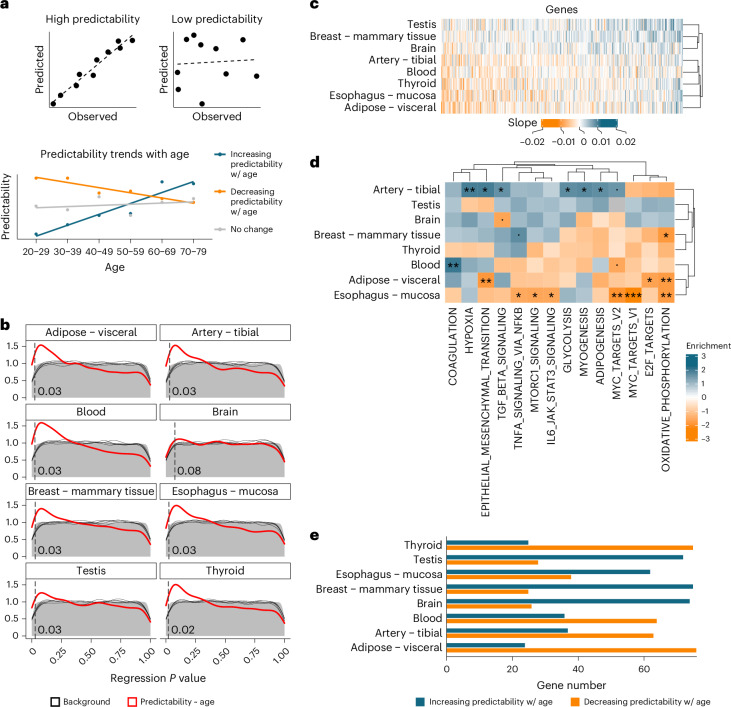
Transcriptome-wide predictability changes with age across tissues. **a**, Computational approach used to identify predictability changes with age. Predictability is quantified as the Spearman correlation between the observed expression patterns (in the original data) and the predicted expression patterns (in the reconstructed data), corresponding to the expected pattern given the expression of regulatory neighbors. A high correlation indicates that the expression pattern of a gene fits the regulatory relationships captured by the model (top left), whereas a low correlation indicates the opposite. Predictability is quantified for groups of samples at six different age groups: the decades spanning 20–29 to 70–79. For each gene, predictability is modeled as a linear function of age. **b**, Distribution of *P* values for the regression of predictability values within each age group against the mean age of the age group. *P* values correspond to the two-sided *t*-test on regression coefficients, without multiple testing correction. Red line: *P* value distribution obtained from the real data. Gray background: average *P* value distribution across 100 permutations of the age groups. Black lines: five individual permutations randomly picked from the background. The dashed vertical line indicates the highest *P* value among the genes considered statistically significant in each tissue (orange and blue bars in **e**). The number of genes included in each tissue-specific analysis can be found in Supplementary Table 3. **c**, Heatmap of the predictability slopes across all 370 genes, independently of significance level, ordered by increasing average predictability across tissues. Only the 370 genes for which the regression analysis was performed in all eight tissues were included—that is, the genes with a high average predictability in all eight tissues (Supplementary Table 3). **d**, Hallmark gene sets enriched in age-related gene–gene relationship changes, captured by GSEA. The heatmap shows all hallmarks with statistically significant (FDR < 0.05) enrichment in at least one tissue. **e**, Number of genes with predictability increase (blue) and decrease (orange) among the top 100 most significant genes per tissue. ***FDR < 0.001; **FDR < 0.01; *FDR < 0.05; ^.^FDR < 0.1. FDR, false discovery rate.

To quantify such changes in regulatory programs, we established a metric of (de)regulation, based on the extent of agreement between the expression profile of a given gene in the data and the reconstructed profile according to its regulatory neighborhood (that is, comparing observed versus predicted expression levels). To quantify this agreement, we used Spearman’s rho, computed between the expected and observed expression of a gene across samples. We refer to this metric as the ‘predictability’ of a gene in each group of samples (Fig. 2a). Our choice of Spearman over Pearson correlation was based on its lower sensitivity to outliers, which avoids that a few individuals with outlying expression of the target gene dictate the overall predictability. To capture age-related changes in predictability, we made use of the wide age range of the donors in the GTEx dataset. We restricted our analysis to the 20 tissues with highest sample number and split the data into six similarly sized age groups, defined as age decades (20–29 up to 70–79; Methods). Splitting the samples by decade resulted in 30–62 samples per tissue and age split (Supplementary Table 1). We then computed the predictability of each gene in every tissue–age split and restricted our analysis to genes showing sufficiently high average predictability across age groups (Methods). This resulted in the selection of 3,291–5,830 genes per tissue (Supplementary Table 3). We then regressed predictability of these genes as a function of age using a linear model (Predictability ~ Age; Fig. 2a). This procedure resulted in one slope for each gene in each tissue, quantifying the change of its predictability with age. To determine the statistical significance of the resulting predictability slopes, we compared the *P* value distribution for the obtained regression slope with the null *P* value distribution, obtained after repeatedly shuffling the age groups (Methods). We observed large differences in the predictability signatures of different tissues, with some tissues showing an enrichment in small *P* values but not others (Extended Data Fig. 5). Notably, we did not observe a clear global trend toward decreasing (or increasing) predictability with age. Although some tissues (for example, Blood and Thyroid) had more genes with decreasing predictability, other tissues had more genes with increasing predictability (for example, Breast – mammary tissue, Nerve and Esophagus – mucosa). We focused our subsequent analysis on eight tissues with substantially more genes showing an age-dependent predictability change than expected by chance—that is, those tissues with an inflation of small *P* values (Adipose – visceral, Artery – tibial, Blood, Brain, Breast – mammary tissue, Esophagus – mucosa, Testis and Thyroid; Fig. 2b and Supplementary Table 1). First, we analyzed predictability changes of 370 genes that could be analyzed across all of those eight tissues, because their average predictability was sufficiently high in all of them (Fig. 2c). When comparing the predictability slopes of those 370 genes, we observed that many of them showed similar predictability changes across multiple tissues. This was especially true for genes with age-related decrease in predictability (Fig. 2c, left). When grouping tissues according to their age-related predictability change profiles, we observed a separation of Testis, Breast and Brain from the remaining tissues, accompanied by a more predominant age-related increase in predictability in these tissues. The distinct predictability changes in these tissues are consistent with earlier analysis of the GTEx cohort revealing regulatory programs distinguishing Brain and Testis from the other tissues^21^.

We next sought to understand which cellular functions were most affected by the observed age-related predictability changes, either commonly across tissues or specifically for each tissue. To this end, we performed gene set enrichment analysis^22^ (GSEA) on the age-related predictability slopes for all genes included in the analysis in each tissue, to identify gene sets with significant enrichment in age-related predictability changes in each tissue (Methods). We focused our analysis on gene sets (Molecular Signatures Database (MSigDB) hallmarks, Fig. 2d, or GO terms, Extended Data Fig. 7) showing a significant enrichment in at least one tissue. This analysis revealed that genes involved in oxidative phosphorylation were among the most affected by predictability changes across several tissues, showing a predictability decrease in all tissues apart from Brain and Testis (Fig. 2d and Extended Data Fig. 7). Targets of the E2F TF family, known to regulate the expression of genes involved in the transition between the G1 and S phases of the cell cycle, and targets of the oncogene MYC, also involved in cell cycle regulation, apoptosis and differentiation, showed the same trend toward decreased predictability in all tissues apart from Brain and Testis. These results suggest age-related changes in gene–gene relationships of cell proliferation and mitochondrial genes.

Other functions differently affected across tissues included metabolism (mTORC1 signaling and glycolysis; Fig. 2d), development (adipogenesis, myogenesis and epithelial–mesenchymal transition, Fig. 2d; axonogenesis and neuron development, Extended Data Fig. 7), cell growth (mTORC1 signaling and TGFβ signaling; Fig. 2d) and immune response (TNF/NF-κB signaling and IL-6/JAK/STAT3 signaling; Fig. 2d). Of note, gene sets with tissue-specific functions also showed age-related predictability changes in the respective tissue. Among these, we highlight the increased predictability of hypoxia genes in Artery and of coagulation genes in Blood (Fig. 2d) and the decreased predictability of synaptic genes in Brain (Extended Data Fig. 7).

To further corroborate those results, we selected in each tissue the 100 genes with the most significant predictability changes with age (lowest regression *P* values), independent of the direction of that change. We termed these genes ‘high-confidence predictability hits’. For those genes, we also observed considerable differences between tissues, with Blood, Thyroid, Artery and Adipose Tissue showing mostly decreases in predictability with age, and Testis, Breast, Brain and Esophagus showing mostly increases in predictability (Fig. 2e). To exclude that these differences between tissues are driven by differences in sample numbers, we repeated our analysis with similar sample numbers (*n* = 30) across tissues and age groups (Extended Data Fig. 5 and Supplementary Table 2), which removed differences between tissues due to different sample sizes. This resulted in statistical significance (Extended Data Fig. 5b) and slope directions (Extended Data Fig. 5d) consistent with those obtained with larger sample sizes. Finally, we also repeated our analysis excluding the age group 70–79, as the smaller number of samples in this group might have influenced the predictability values and skewed the regression slope (Supplementary Table 1). However, we once again observed consistent statistical significance (Extended Data Fig. 6b) and preference toward positive or negative slopes (Extended Data Fig. 6d) upon exclusion of this age group. Taken together, these results suggest that predictability is affected by age in some tissues more than others and that the direction of this effect varies between tissues.

To confirm these findings in an external dataset unaffected by tissue composition changes, we computed age-related predictability changes in scRNA-seq data from PBMCs of 982 human donors^20^. We computed age-related slopes across all cell types as well as per cell type (Methods) and compared to the age-related slopes previously obtained in GTEx blood. This comparison revealed an agreement between high-confidence predictability hits found in GTEx blood and those found in T cells (Extended Data Fig. 8), suggesting that the tissue-level age trends may be driven by T cell age trends instead of age-related changes in cell type composition.

### Regulatory relationship changes with age

**Fig. 3:**
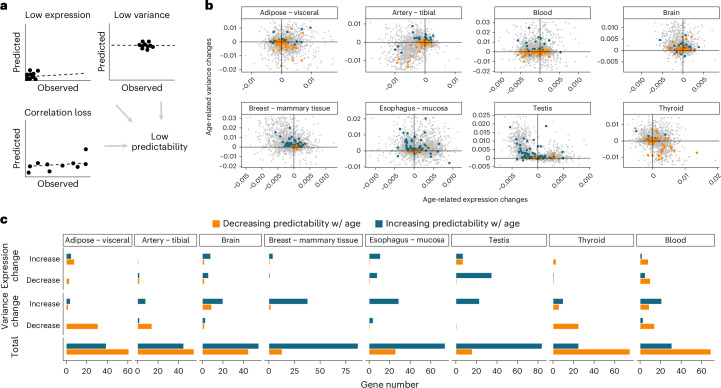
Factors underlying age-related predictability changes. **a**, Possible factors explaining observations of low predictability and resulting comparisons of observed values (in the original data, *x* axis) versus predicted values (data reconstructed with our regulatory model, *y* axis). Top left, low average expression of the target gene results in noisy quantifications. Top right, low variance of the target gene results in almost constant expression values. Correlation approaches thus capture noisy fluctuations around the mean. Bottom left, loss of correlation with the regulatory neighborhood results in a failure of the model to correctly capture the expression pattern of the target gene. **b**, Expression slope across age (*x* axis) against variance slope across age (*y* axis) for background genes (small light gray points). Top 100 significant genes with predictability increase (large blue points) or decrease (large orange points) with age. Age-related variance changes are cropped to remove outliers (see Extended Data Fig. 9a for full data). **c**, Number of genes with predictability decrease (orange) or increase (blue) in each of the three scenarios tested: ‘Expression change’, ‘Variance change’ or ‘Total’ (total number of predictability hits, for reference).

Next, we sought to analyze the causes underlying age-associated predictability changes. By definition, low predictability can result from changes in the structure of the regulatory model itself—that is, if the regulatory relationships captured by our model are not met (Fig. 3a, ‘Correlation loss’). However, other factors can modulate predictability. One such factor is the average expression level of the gene at hand: if average gene expression levels are low, the quantification becomes noisier (fewer reads per gene), resulting in lower predictability (Fig. 3a, ‘Low expression’). Another factor is gene expression variance, as a small range of expression values impairs correlation quantification and, thus, predictability (Fig. 3a, ‘Low variance’).

First, we quantified average expression level and variance changes with age. We observed a clear trend toward variance increase with age across all tissues except Artery and Thyroid (Fig. 3b, *y* axis), in line with earlier reports of increased inter-individual transcriptomic variability with age^23,24^. The direction of average expression changes with age varied across tissues, with Artery, Brain and Breast showing mostly expression decrease with age, Thyroid showing mostly expression increase with age and the remaining tissues showing similar expression changes in both directions (Fig. 3b, *x* axis). We found the quantification of average expression and variance changes with age to be influenced by the 70–79 age group in some tissues (Fig. 3b versus Extended Data Fig. 9b). For this reason, the process described below for the identification of genes with predictability, average expression or variance changes is limited to genes showing the same trend when including and excluding the 70–79 age group (Methods).

To quantify the contribution of average expression and variance changes to age-associated predictability changes, we compared age-related changes in each of these factors with age-related predictability changes. We applied a cutoff on the absolute values of the expression fold changes or variance slopes with age (0.001) and then counted the number of high-confidence predictability hits (either increase or decrease in predictability) that also showed age-related changes in expression levels (Fig. 3c, ‘Expression changes’) and in expression variance (Fig. 3c, ‘Variance changes’).

We observed relatively few genes whose age-associated expression changes were linked to predictability changes (Fig. 3c, ‘Expression change’), suggesting that age-related differences in average expression levels do not explain the predictability changes captured by our approach. Genes with increased variance with age almost always increased their predictability, and, conversely, genes with decreased variance mostly decreased their predictability with age (Fig. 3c, ‘Variance change’), suggesting that age-related changes in gene expression variance influence changes in predictability.

Taken together, our analyses suggest that predictability changes captured by our regulatory model are rarely explained by age-related changes in average expression levels. Instead, predictability changes are, in part, explained by changes in inter-individual variability.

**Fig. 4:**
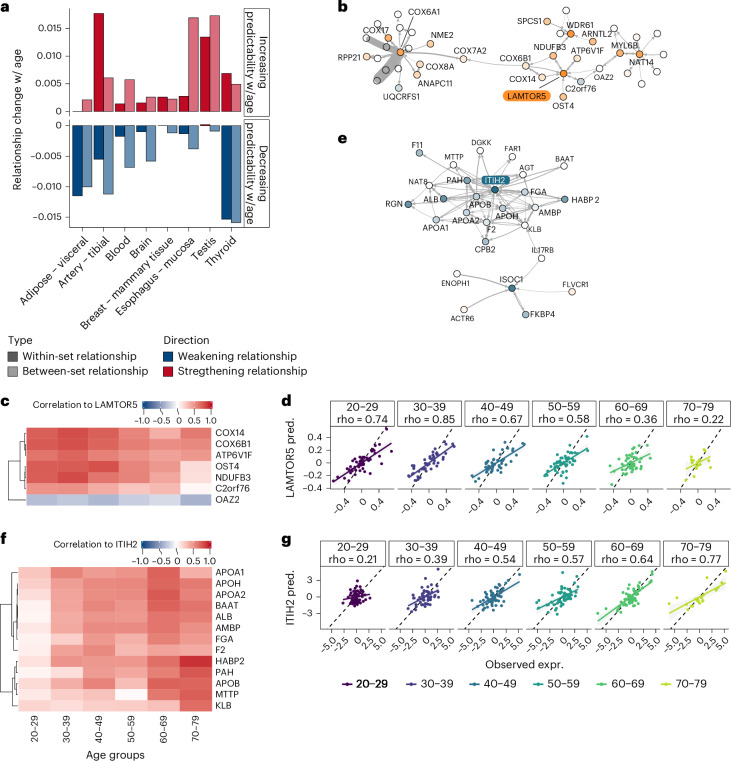
Within-set and between-set regulatory relationships altered with age. **a**, Contribution of within-set and between-set correlation changes to age-related predictability changes, represented as the weighted sum of age-related correlation changes between genes with increasing (top) or decreasing (bottom) predictability with age and their regulatory neighbors within (full color) or between (transparent) the same gene set. Correlation changes with age are quantified as the slope of the correlation ~ Age regression. These values are weighted with the coefficients of the regulatory model, so that correlation changes in strongly connected regulatory neighbors (higher coefficient) are prioritized. **b**,**e**, Subnetwork of the neighborhood of genes with age-related predictability changes. Nodes represent genes, colored by predictability slope in the respective tissue. Connections between nodes represent gene–gene relationships captured by the regulatory model. **b**, Neighborhood of LAMTOR5, colored by predictability slope with age in Artery – tibial. **e**, Neighborhood of ITIH2, colored by predicatability slope with age in Blood. **c**,**f**, Correlation between expression of genes with predictability changes (LAMTOR5 in **c** and ITIH2 in **f**) and regulatory neighbors across age groups. **d**,**g**, Comparison of expression of genes LAMTOR5 (**d**) and ITIH2 (**g**) in the original data (observed expression, *x* axis) and reconstructed expression based on the regulatory neighborhood (predicted expression, *y* axis). The linear regression fit to the trend is shown along with the 95% confidence interval bands. pred., predicted.

We next assessed whether correlations to neighbors involved in the same cellular process (MSigDB hallmark) and to neighbors in different cellular processes had a similar impact on the observed changes in predictability. To this end, we grouped the neighbors of each predictability hit according to their functional annotation and summarized age-related correlation changes (Methods) across all neighbors in the same process. This analysis was performed separately for targets with increased and decreased predictability. We then compared the total contribution of within-set relationships (that is, age-related correlation changes between a hit and a neighbor in the same process) and between-set relationships (that is, age-related correlation changes between a hit and a neighbor in a different process). For decreasing predictability hits, we observed that, across all tissues except Adipose, between-set relationships had more impact on predictability changes than within-set relationships (Fig. 4a, bottom). For all tissues except Breast, Esophagus and Testis, between-set relationships had more impact on age-related predictability decreases compared to increases (Fig. 4a, top versus bottom). To take into account differences in the number of neighbors between gene sets, we repeated our analysis averaging the contributions across all regulatory neighbors of the same gene set (weighted average according to the weights in the regulatory model) and found a similar trend toward within-set relationships contributing more strongly toward increasing predictability (Extended Data Fig. 10a). Our results are consistent with a weakening coordination of different functional modules with age, coupled with the strengthening of specific functional responses.

To further explore the link between predictability and within-set/between-set relationship changes, we focused on the subnetworks created by the 20 strongest (highest absolute value of age slopes) predictability hits in each tissue and their immediate regulatory neighbors. We observed both disconnected subnetworks, where one or very few genes showed strong predictability changes with age (exemplified in Extended Data Fig. 10b), and larger subnetworks connecting several genes with strong predictability changes (exemplified in Fig. 4b,e). We then selected for further analysis two of the obtained subnetworks, showing distinct age-related behaviors and each captured in a different tissue.

#### Between-module gene–gene relationship changes with age

One of the subnetworks captured by our analysis (Fig. 4b) was centred on mTOR signaling, which is a well-known contributor to age-related phenotypes and is involved in lifespan extension^25,26^. This subnetwork included the predictability hit LAMTOR5, a gene encoding for a member of the Ragulator complex, located in the lysosomal membrane and involved in mTORC1 activation upon nutrient sensing. In nutrient-rich conditions, mTORC1 is recruited to the lysosomal membrane^27^, resulting in the promotion of cellular growth and proliferation, coupled to translation and biosynthesis^28^. In our analysis, LAMTOR5 showed a loss of correlation with most of its regulatory neighbors in Artery – tibial: cytochrome C oxidases COX14 and COX6B1, NADH dehydrogenase complex member NDUFB3, vacuolar ATPase cytosolic (V1) domain subunit ATP6V1F and oligosaccharyl transferase complex subunit OST4 (Fig. 4c). This loss of correlation took place in parallel with a decrease in predictability with age (Fig. 4d).

Interestingly, also in Artery – tibial, we observed age-related changes in the predictability of another vacuolar ATPase (v-ATPase) subunit (in the transmembrane domain; Extended Data Fig. 10b), also linked to another Ragulator complex member, LAMTOR1. v-ATPases are located on the membrane of multiple organelles, including the lysosome, where they pump protons into the lumen and acidify it^29^. The observed relationships between Ragulator subunits (LAMTOR1 and LAMTOR5) and v-ATPase subunits in both the transmembrane and cytosolic domains (ATP6V1F, ATP6V0D1, ATP6V0C, ATP6V1H, ATP6V1D and ATP6V1A) are consistent with the fact that v-ATPases are required for mTORC1 activation by amino acids^30^. Furthermore, v-ATPases have been described to physically interact with the Ragulator complex through LAMTOR1 (ref. ^30^), in line with the relationship observed between this gene and multiple of the v-ATPase subunits.

In addition, we observed tight gene–gene relationships between Ragulator complex members and electron transport chain members (NDUFB3, COX14 and COX6B1 as direct neighbors of LAMTOR5; UQCRFS1, COX6A1, COX8A, COX17 and COX7A2 as indirect neighbors). These observations are in line with previous work showing that mTORC1 promotes the expression of mitochondrial regulators, such as TFAM and electron transport chain components^31^. These gene–gene relationships were also altered with age, as exemplified by the age-related loss of correlation between LAMTOR5 and its direct electron transport chain neighbors NDUFB3, COX14 and COX6B1 (Fig. 4c).

#### Within-module gene–gene relationship changes with age

Another subnetwork captured by our analysis was composed of serum-specific proteins, most of which showed increased predictability with age in Blood (Fig. 4e). This subnetwork included serum albumin ALB; apolipoproteins APOA1, APOA2, APOB and APOH, involved in lipid transport between tissues; members of the coagulation cascade F2 (thrombin), FGA (a subunit of fibrinogen) and F11 (coagulation factor XI); inter-alpha-trypsin inhibitor chains ITIH2 and AMBP; and serine protease HABP2, involved in hyaluronic acid binding. Serum albumin has been reported to have anticoagulant action^32^, and high-density lipoproteins (HDLs)— composed of APOA1 and, to a lesser extent, APOA2 and APOE—have been shown to modulate platelet reactivity and protect against thrombosis^33^. Additionally, inter-alpha inhibitor activity is required for the formation of the protective hyaluronan coat surrounding some cell types^34^. Hyaluronan has been shown to activate thrombin by blocking its inhibitor antithrombin^35^, supporting the relationships observed between these genes and suggesting a central role of the coagulation cascade in this subnetwork.

We observed an age-related increase of ITIH2 predictability (Fig. 4g), in parallel with an increased correlation to its regulatory neighbors (Fig. 4f), and increased variance across individuals. Inter-alpha inhibitors suppress pro-inflammatory responses and have been shown to improve the outcome of ischemic stroke^36^, which may point toward an adaptive response to an age-related increase in inflammation. This is in line with previous reports of increased blood coagulation potential during aging^37^, including in centenarians^38^.

Taken together, our analyses uncovered gene–gene relationships that seem to be affected by the aging process. The weakening of gene–gene relationships among mTOR regulators, v-ATPase subunits and electron transport chain members may suggest an age-related de-coupling of these processes, which should be coordinated to ensure appropriate response to cellular growth cues. On the other hand, the strengthening of gene–gene relationships between diverse serum proteins may reflect an age-related increase in the coordination of the coagulation cascade and an adaptive response to increased inflammation.

## Discussion

Our regulatory model successfully captures gene–gene relationships that explain the expression pattern of individual genes based on the expression of their regulatory neighborhood (Fig. 1). Notably, our model was able to capture tissue-specific and age-specific differences in relationships between genes involved in the same function, as well as genes involved in different functions, across 30 healthy human tissues^16^. At first, it may seem surprising that our model predicts tissue-specific gene co-expression patterns, because the model itself was not adapted to the tissue. The explanation for this finding is as follows. Whereas the structure of the regulatory network remains largely invariant across tissues, predictors (that is, regulatory neighbors) have different expression levels in different tissues. As a consequence, predicted expression levels of their targets will also be tissue specific. To test to what extent cell-type-specific networks might further improve the predictive power of such models, we trained a blood-specific network on single-cell PBMC data. Genes that were better predicted by the blood-specific network tended to be enriched for cell-type-specific functions, whereas genes that were better predicted by the in vitro cell (cell line)-based data were enriched for basic cellular functions (Extended Data Fig. 2). One drawback of the blood-specific network was its substantially lower gene coverage. Furthermore, PBMCs do not cover all cell types present in whole blood. Thus, coverage of more cell types might further improve the predictive power of such a network. As larger human single-cell transcriptomics datasets with greater technical sensitivity become increasingly available, it may in the future be possible to train cell-type-specific networks that outperform generic networks as the one presented here. However, this does not change the fact that stoichiometric requirements constrain many gene–gene relationships across cell types and tissues.

Based on this regulatory model, we were able to quantify age-related changes in the relationship between individual genes and their regulatory neighbors. Our approach has the advantage of providing such a score for each gene, unlike global approaches based on the whole transcriptome. Additionally, by using robust gene–gene relationships trained on external data, our model captures the relationships underlying a vast range of different regulated states of the cellular transcriptome (that is, the transcriptomic states of different cancer cell lines). Thus, our metric of predictability corresponds to the extent to which the gene–gene relationships of a given gene fit those captured in the training data. This is conceptually different from, and complementary to, a differential co-expression analysis, where gene–gene relationships are compared in two conditions (old versus young), without any external information. It should be noted that our regulatory model reflects only a part of the complete space of gene–gene relationships that can take place. In particular, the captured relationships are those that are common between the GTEx dataset and the different cancer cell lines in the training dataset. This means that we cannot rule out the possibility that observations of low predictability come from a gain in correlation with different regulatory neighbors that were not captured by the training data rather than a complete loss of regulatory control. In any case, a change in predictability reports on a change in gene expression regulation.

Using the regulatory relationships captured in our model to quantify changes in expression coordination (predictability), we observed substantial difference in the aging patterns of different tissues. Not all tissues showed significant changes in predictability (Extended Data Fig. 1b), and, among those that did, the direction of the global predictability trends sometimes differed between tissues. It is important to note that changes in the signal strength between tissues may originate from differences in data quality or confounding factors that we could not fully correct for, despite our pre-processing efforts. Additionally, we cannot exclude a possible impact of age-related changes in nonlinear relationships between genes, which may not be correctly captured with our model.

Two major observations of our analyses are the following. First, we did not observe a substantial decline in predictability with age across tissues. Instead, predictability changes happened in both directions and were highly tissue dependent. Second, increasing inter-individual variation of gene expression was often associated with increasing predictability. Hence, increasing expression divergence resulting, for example, from different lifestyles is the result of regulated gene activity changes. Taken together, these findings show that aging does not lead to a global deregulation of gene expression—that is, simply ‘noisier’ expression—at least not at the bulk tissue level.

Predictability changes that were consistent across tissues mostly linked to mitochondrial functions and cell cycle regulation/proliferation. Mitochondrial dysfunction is an established hallmark of aging^1^, and a decrease in ATP production has been reported with age^39^, along with alterations in mitochondrial morphology and mitochondrial protein expression^40^. In parallel to age-related deregulation of mitochondrial functions, we also observed a deregulation of genes involved in cell cycle regulation, proliferation and development (Fig. 2d and Extended Data Fig. 7). We note that our regulatory model has been trained in cancer cell lines (proliferating, often with mutations in the tumor suppressor p53 and oncogene MYC), which requires some caution when analyzing results concerning genes involved in cell cycle regulation and cellular proliferation. Our analysis of age-related predictability changes only includes genes with high average predictability across age groups, which excludes genes whose regulatory relationships are exclusively valid in the context of cancer data but not healthy tissues. Note that genes that changed their predictability with age were generally not enriched in cancer-specific functions and included tissue-specific functions (Extended Data Fig. 7). Second, the analysis captured changes in relative predictability across aging within one tissue. Those within-tissue predictability changes are independent of tissue-to-tissue variation in predictability. This notion is further supported by the fact that global trends in predictability changes did not seem to depend on how ‘proliferative’ a tissue was; compare, for example, Brain versus Breast or Esophagus in Fig. 2e. Finally, energy metabolism was also affected by age-related predictability changes across a diverse range of tissues. Energy metabolism is known to have widespread consequences in the cellular transcriptome beyond the direct control of energy production^41,42^. In fact, a coordinated balance of distinct cellular functions, such as glycolysis and oxidative phosphorylation, is required. Accordingly, we found both glycolysis and oxidative phosphorylation to be affected by age-related loss of predictability, along with mTORC1 signaling. This observation raises the question of whether aging affects the coordination between these different cellular modules or, rather, the coordination of gene expression within each module.

Our approach has the advantage of capturing gene–gene relationships both within and between different functional gene modules (Fig. 4a). We found weakening gene–gene relationships to be mostly between different functional modules, whereas strengthening gene–gene relationships spanned both within-module and between-module relationships. The age-related loss of coordination between cellular processes was exemplified in Fig. 4b, for a subnetwork of genes centered on mTOR signaling and in line with the results discussed previously. The variety of processes captured in this subnetwork is in line with the known mechanisms of mTORC1 activation and its role in the regulation of cellular growth, which requires the coordination of distinct cellular processes. In this subnetwork, the connection between mTORC1 activation and mitochondrial functions was particularly evident. The observed loss of correlation between all these interconnected cellular processes suggests that, with age, cells are no longer able to coordinate different processes to put in place an integrated, complex response, such as cellular growth. This result is particularly interesting in the context of the role of mTOR signaling in aging and longevity. Inhibition of mTOR with rapamycin is well known to extend lifespan across species^43,44^, and an increase in mTOR activation has been reported with aging in some tissues^45^. Our results suggest a decoupling between the mechanistic activation of mTORC1 and some of its downstream target pathways that may be linked to an inability of the cell to sustain cellular growth. In fact, chronic activation of mTORC1, as observed during aging, is known to be detrimental to health^46^ and has been shown to lead to, for instance, pancreatic β cell death due to the accumulation of damaged organelles^47^. As an example of age-related strengthening of gene–gene relationships within a functional module, we focused on the increased predictability observed in the coagulation network in Blood (Fig. 4e). Genes with increased predictability strengthened their relationships to regulatory neighbors and showed increased variance between individuals with age, in line with a regulated, adaptive response to the aging process, with different levels of activity in different individuals.

Finally, we note that we used bulk RNA-seq data, which consist of a mixture of the transcriptome of the different cells present within the tissue. Thus, our approach cannot capture transcriptional noise resulting from random expression in individual cells. In our case, such noise would be averaged out across the different cells in the tissue. This is in line with the fact that we barely observed any genes with increased variance and decreased predictability with age (Fig. 3b). We hypothesize that the gene–gene regulation changes that we found may, in part, be driven by age-related alterations in the cell type composition of the tissues or by a rewiring of the relationships in one leading cell type within the tissue. We capitalized on PBMC scRNA-seq data to compute predictability changes for the major PBMC cell types and found that aggregated (cross-cell-type) data and the T cell data resulted in predictability changes that were consistent (positively correlated) with the GTEx-derived effects. This is consistent with the notion that, among the PBMC cell types tested, T cells were dominating the blood signal in the GTEx data. In conclusion, even though some of the effects that we observed might be due to cell composition changes, the effects are at least partially due to cell-intrinsic changes in gene expression, because they are reproducible in ‘pure’ cell types. As scRNA-seq data from multiple human tissues continue to accumulate, it will become possible to further quantify age-related changes in predictability in a cell-type-specific manner and elucidate the contribution of different cell types to tissue-level changes.

Our work highlights the importance of zooming out of the effect of aging in individual genes or cellular processes and investigating how their crosstalk is affected at a systems level. Given the complex and multifactorial nature of aging, we expect that approaches such as the one presented here will contribute to a better understanding of the global alterations in functional coordination that take place with age.

## Methods

### Statistics and reproducibility

Analyses were conducted in R version 4.0.3. Only publicly available data were used; sources are given above. Therefore, we had no influence on the study design or sample numbers. We always aimed to use the maximum number of samples available. Criteria for excluding samples are given below (see the ‘Subsetting’ subsection).

### Gene regulatory network training

The gene regulatory network (GRN) was trained as previously described^16^. A combination of regularized linear regression and stability selection was used to identify genes whose expression pattern is informative (predictive) of the expression pattern of a given gene. The expression pattern of each gene was then modeled as a linear function of the expression patterns of informative genes. This procedure was applied to 1,376 cancer cell lines with known karyotypes^48,49^. The weights of this linear model, learned in the cancer cell line data, capture the direction and strength of the relationship between the predictor and target genes.

### Network processing

First, residual edges were removed. This was done by computing, for each edge pair (i,j) (j,i), the ratio of between the lowest and the sum of the absolute edge weights. When this ratio was below 0.1, the weakest edge (lowest absolute value) was removed from the network. To avoid effects captured by the network, which are not due to regulatory interactions between genes but, rather, local effects (for example, both genes impacted by a copy number change in the training data), we removed predictors located in the same chromosome arm as the target. The largest connected component of the resulting network was then determined using the function components() from the igraph R package (version 1.2.6), with default parameters. All subsequent analyses were performed on the largest connected component.

### Edge density analysis for TF target genes

Sets of genes regulated by the same TF were retrieved from the Dorothea database^19^, and publicly available TF targets were inferred from different GTEx tissues^18^. The org.Hs.eg.db R package was used to map the Entrez gene IDs to gene symbols. The edge density of each gene set was quantified using the edges of the network trained on cancer cell line data. For each gene set, edge density was computed for the subnetwork defined by the genes within the gene set and compared to the edge density of the full network. The edge density for a gene set was defined as the fraction of nonzero edges within that set, normalized by the maximum possible number of edges: ${k}_{i}\times \frac{{k}_{i}-1}{2}$, where *k*_*i*_ represents the number of genes in gene set *i*. One-sample *t*-tests were conducted to statistically assess the edge densities against the background edge density of the entire network. The null hypothesis posited that the edge densities of the gene sets are equivalent to the background density.

### GTEx data pre-processing

#### Data download

Read counts were downloaded from the GTEx portal (version 8).

#### Data pre-processing

Genes were filtered based on biotype and average expression across the whole dataset. Biotypes were limited to protein coding, lincRNA, snRNA, miRNA and snoRNA. Genes with average read count below 100 across the whole dataset were excluded.

Filtered data were normalized using DESeq2 version 1.30.1. Sample size factors were estimated with the function estimateSizeFactors(), and normalization was done with the counts() function, with normalized = TRUE. Normalized expression levels were log transformed (base 2 with pseudocount of 1).

To regress out the effect of confounding variables on gene expression levels, expression levels across samples of each tissue (SMTSD) were modeled as a function of ischemic time (SMTSISCH), batch (SMGEBTCH), Hardy scale (DTHHRDY) and sex (SEX), using base R’s lm() function. For Testis and Breast samples, sex was left out of the linear model. The residuals of the linear regression were used as batch-corrected data.

#### Subsetting

To assure the same number of samples in each age group, each age group was downsampled to the size of the smallest age group (determined excluding the 70–79 group). Samples from different regions of the brain were grouped into the same tissue by selecting, for each subregion in each age group, five samples. For groups of similar regions (cerebellar cortex and cerebellum, different regions of the cortex and different regions of the basal ganglia), three samples from each of the subregions were picked instead of five. The selection of the three or five samples per region and age group was done in a way that maximizes sampling across different donors. Within each age group, donors with the least available brain samples (across all regions) were prioritized, and, after each round of sample selection (one round per region), the selected donors acquired the lowest priority. The age group 70–79 was included provided that at least ten samples were available.

To assure the same number of samples for each tissue (SMSTD) and in each age group (same-sized subset analyses; Fig. 1, Extended Data Fig. 5 and Supplementary Tables 2 and 4), 30 samples were randomly selected per age group. Brain samples were handled in the same way as described above, with the exception that three samples were chosen per region and age group, except for regions in groups of similar regions, for which two samples were chosen per age group. The age group 70–79 was included provided that at least ten samples were available. Cross-tissue samples were generated by combining two samples of each one of the 16 tissues analyzed, leading to a total of 32 samples per age group.

### Expression profile reconstruction using the regulatory model

The training procedure in the cancer cell line data resulted in a set of robust predictors and respective weights for each gene. To reconstruct profiles based on the regulatory relationships captured by the model, the expression data were centered at 0 for each gene, and the relative expression pattern of the predictor genes was multiplied by the respective weights from the regulatory model.

### Identification of poorly predicted genes

**Extended Data Fig. 4:**
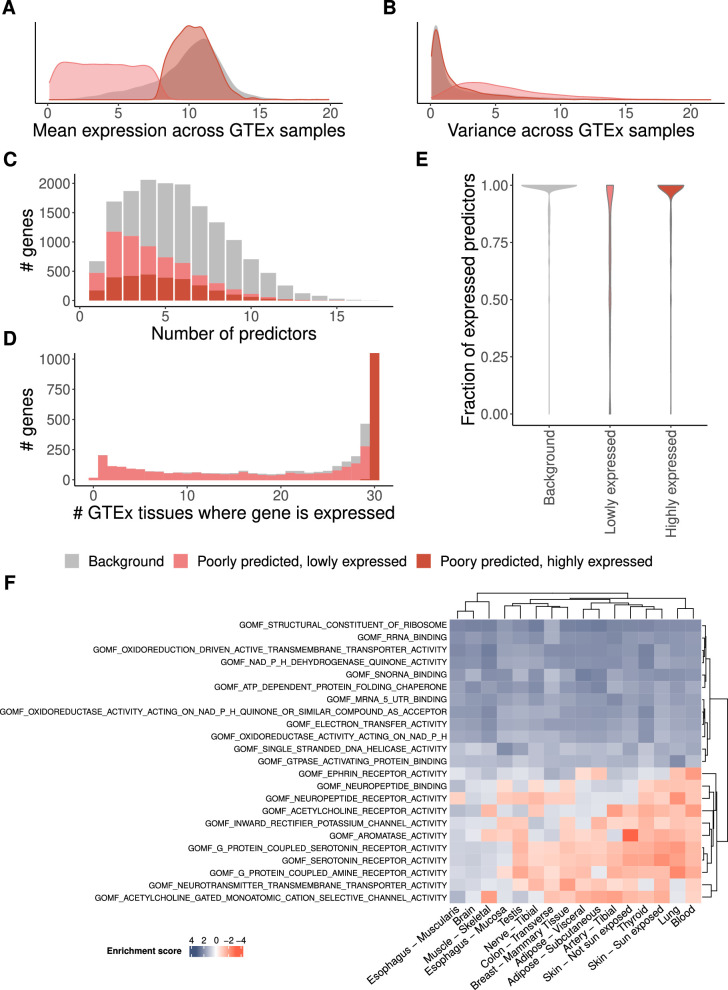
Characterization of genes poorly predicted by the regulatory model. Poorly predicted genes were defined as having an average Spearman’s rho across all 30 tissues < 0.2 and split into 2 groups according to their average expression levels across GTEx tissues: lowly expressed genes (light pink) and highly expressed genes (dark orange). **a)** Average expression levels across all GTEx samples. **b)** Expression variance across all GTEx samples. **c)** Histogram of number of predictors (genes with expression profiles informative for the target gene). **d)** Histogram of number of tissues where the target gene is expressed (average normalized expression within tissue > 2). **e)** Distribution of the fraction of predictors of a given target that are expressed (average normalized expression across tissues > 2). Histogram bars in C and D are stacked. **f)** Functional analysis of well vs poorly predicted genes. Genes were ordered based on their predictability in each tissue and a Gene Set Enrichment Analysis was performed using molecular function GO terms. Heatmap colors correspond to normalized enrichment scores. The heatmap shows all terms with statistically significant (FDR < 0.05) enrichment in at least one tissue.

**Extended Data Fig. 5:**
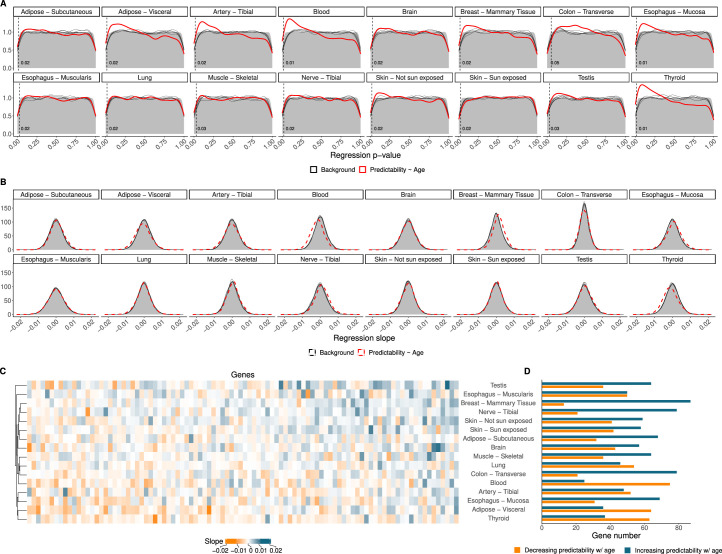
Transcriptome-wide predictability changes with age: expanded for all 16 tissues. Across all tissues, age groups were downsampled to 30 samples. The age group 70–79 was included in the analysis, with as many samples as were available. For sample size details see Supplementary Table 2. **a, b)** Distribution of p-values (**a**) and slopes (**b**) for the regression of predictability values within each age group against the mean age of the age group. Red line: p-value or slope distribution obtained from the real data. Grey background: average p-value or slope distribution across 100 permutations of the age groups. Black lines: 5 individual permutations randomly picked from the background. **a)** The dashed vertical line indicates the highest p-value among the genes considered statistically significant in each tissue (orange and blue bars in D). The number of genes included in each tissue-specific analysis can be found in Supplementary Table 4. P-values correspond to the two-sided t-test on regression coefficients, without multiple testing correction. **c)** Heatmap of the predictability slopes across all genes, independently of significance level, for which the regression analysis was performed in all 16 tissues (96 genes). These genes had a high average predictability in all 16 tissues (Supplementary Table 4). **d)** Number of genes with predictability increase (blue) and decrease (orange) among the top 100 most significant genes per tissue.

**Extended Data Fig. 6:**
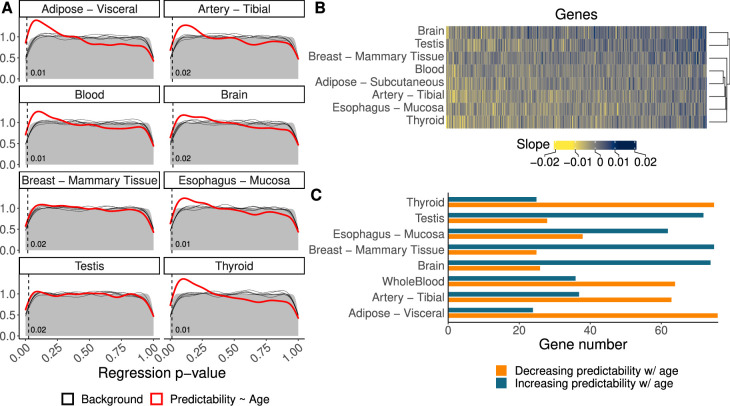
Transcriptome-wide predictability changes with age: excluding the age group of 70–79. **a)** Distribution of p-values for the regression of predictability values within each age group against the mean age of the age group. Red line: p-value distribution obtained from the real data. Grey background: average p-value distribution across 100 permutations of the age groups. Black lines: 5 individual permutations randomly picked from the background. The dashed vertical line indicates the highest p-value among the genes considered statistically significant in each tissue (orange and blue bars in C). P-values correspond to the two-sided t-test on regression coefficients, without multiple testing correction. **b)** Heatmap of the predictability slopes across all genes, independently of significance level, for which the regression analysis was performed in all 8 tissues. These genes had a high average predictability in all 8 tissues. **c)** Number of genes with predictability increase (blue) and decrease (orange) among the top 100 most significant genes per tissue.

**Extended Data Fig. 7:**
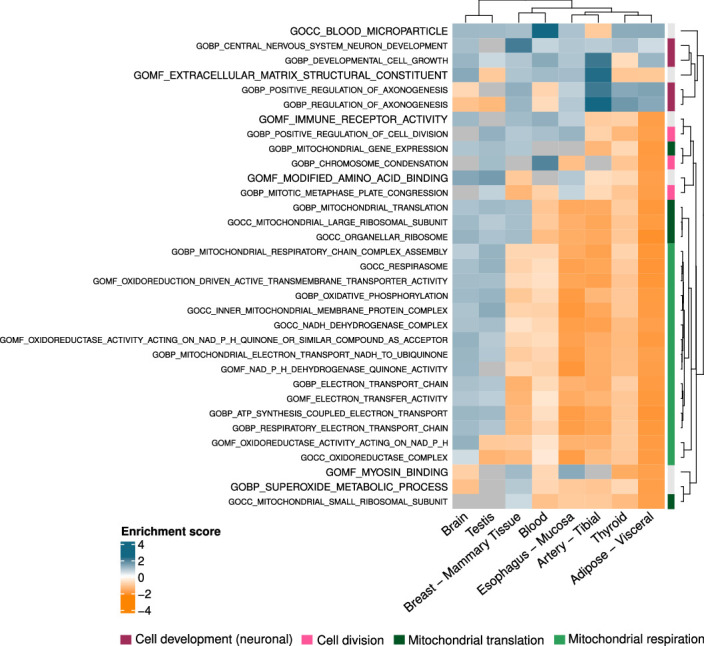
GO terms enriched in age-related gene-gene relationship changes, captured by Gene Set Enrichment Analysis. Genes were ranked by their predictability change with age (top ranking genes: strongest positive change, bottom ranking genes: strongest negative change). The heatmap shows all GO terms with statistically significant (FDR < 0.05) enrichment in at least one tissue at the two ends of the scale.

**Extended Data Fig. 8:**
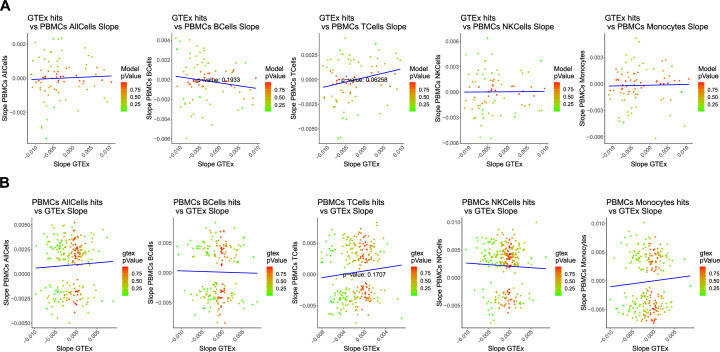
Comparison of age-related predictability changes in the GTEx and PBMC datasets. **a)** Comparison of age-related slopes of GTEx Blood high-confidence predictability hits in the GTEx dataset (x-axis) *versus* the age-related slope of the same genes in the PBMC dataset (y-axis). **b)** Comparison of age-related slopes of predictability hits in the PBMC dataset in the GTEx dataset (x-axis) *versus* the PBMC dataset (y-axis). Dots correspond to individual genes and are colored according to the p-values of predictability changes within either the PBMC dataset (**a**) or the GTEx dataset (**b**). Hits are defined as genes with significant (p-value < 0.05) age-related predictability changes in the GTEx dataset (**a**) or the PBMC dataset (**b**). Each panel represents a different PBMC cell type, with a collective analysis (pooled cell types) in the first and individual cell types (B cells, T cells, NK cells, Monocytes) in the others. A linear trend line (blue) was fit on each panel, nearing significance for GTEx hits in T cells within the PBMC dataset (panel A, p-value = 0.063).

**Extended Data Fig. 9:**
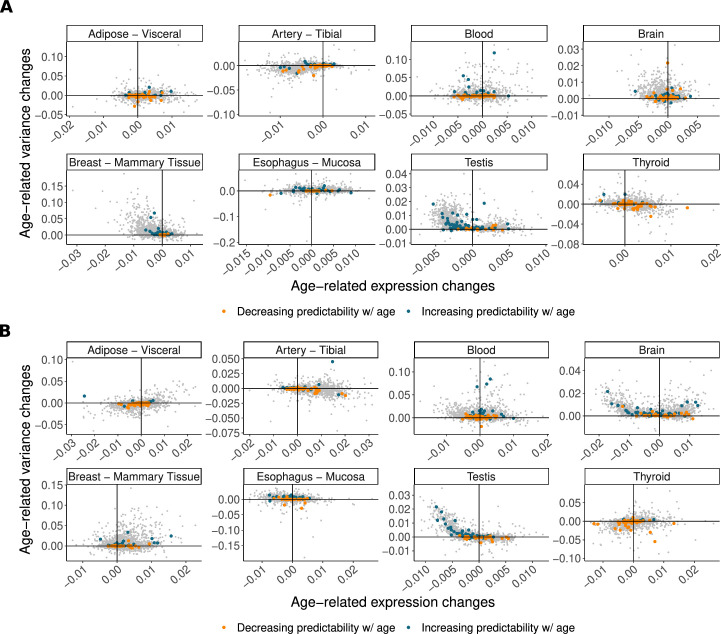
Expression slope across age (x-axis) against variance slope across age (y-axis. **a)** Including the 70–79 age group. **b)** Excluding the 70–79 age group. Background genes are represented as small light grey points and top 100 significant genes with age-related predictability changes as large colored points (predictability increase in blue and decrease in orange).

**Extended Data Fig. 10:**
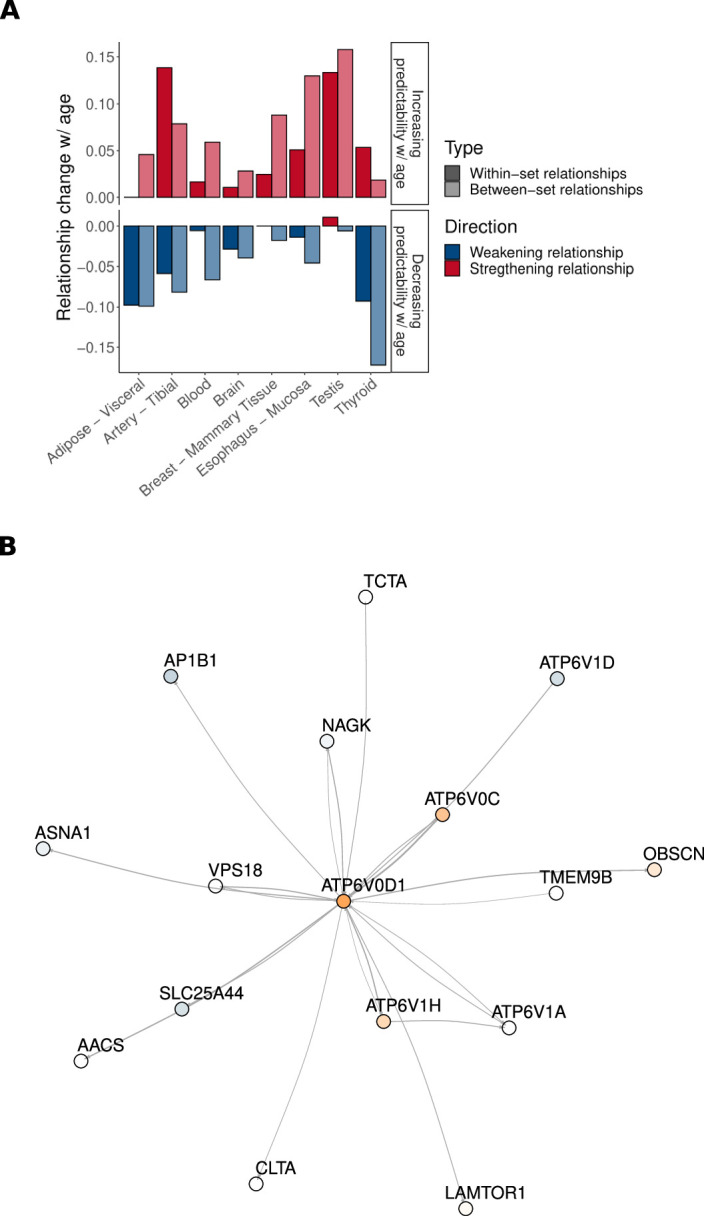
Contribution of within-set and between-set correlation changes to age-related predictability changes. **a) Contribution of within-set and between-set correlation changes to age-related predictability changes**. Predictability changes are represented as the weighted averages (opposed to weighted sums in Figure 4a) of age-related correlation changes between genes with increasing (top) or decreasing (bottom) predictability with age and their regulatory neighbours within (full color) or between (transparent) the same gene set. Correlation changes with age are quantified as the slope of the correlation ~ Age regression. These values are weighted with the coefficients of the regulatory model, so that correlation changes in strongly connected regulatory neighbours (higher coefficient) are prioritized. **b) Regulatory neighbourhood of ATP6V0D1**. This gene encodes for a subunit of the transmembrane (V0) domain of vacuolar ATPase. Nodes represent genes, colored by predictability slope in Artery - Tibial. Connections between nodes represent gene-gene relationships captured by the regulatory model.

Poorly predicted genes (Extended Data Fig. 4) were identified based on the Spearman correlation between the observed expression levels (original data after normalization and batch correction) and the predicted expression levels (reconstructed data based on the regulatory model). Genes with average Spearman correlation coefficient across tissues below 0.2 were identified as poorly predicted. Functional characterization of genes based on their predictability was performed using GSEA^22^ version 4.2.3 for Linux in PreRanked mode. For each tissue, genes were ranked according to the Spearman correlation coefficient between observed and predicted expression levels. The MSigDB^50^ (version 2022.1, updated in August 2022) was used to retrieve GO (Molecular Functions) gene sets.

### PBMC data pre-processing

#### Data download

PBMC processed data from Yazar et al.^20^ were loaded into an R environment using the Seurat package. Metadata annotation, including age and sex, was integrated into the Seurat object.

#### Data pre-processing

Quality control metrics were visualized to assess the distribution of features, such as mitochondrial content, number of detected genes and total counts per cell. It was decided to accept the quality control already done by the authors. Normalization was performed using Seurat’s NormalizeData() function.

#### Cell type reannotation and dataset refinement

Existing cell type annotations were combined to broader cell type categories to increase the sample numbers per cell type. Specific subtypes of T cells, including central memory CD4^+^ cells, αβ T cells and effector memory CD8^+^, αβ T cells, were reclassified under the broad category of ‘T Cells’. Similarly, natural killer (NK) cells and CD16^−^, CD56-bright NK cells were grouped as ‘NK Cells’. Monocyte subtypes, such as CD14-low, CD16^+^ monocytes and CD14^+^ monocytes were grouped as ‘Monocytes’, and naive B cells, memory B cells and transitional stage B cells were grouped as ‘B Cells’. Additional groupings were made for ‘Plasmablast’, ‘Erythrocytes’, ‘Platelets’, ‘HPCs’, ‘Dendritic cells’, ‘Thymocytes’, ‘Lymphoid cells’ and ‘PBMCs’, which encapsulated their respective subtypes. After reannotation, subsets of the data were created by excluding cell types with an average of fewer than 40 cells per donor. After this filter, T cells, B cells, NK cells and monocytes were kept for subsequent analyses.

#### Dimensionality reduction and integration

Canonical correlation analysis integration was performed using the Seurat R package. In total, 2,000 integration features were identified using SelectIntegrationFeatures(). These features were used to find integration anchors using FindIntegrationAnchors(), and the data were subsequently integrated using those anchors as inputs to IntegrateData(). The integrated data were subsequently scaled, centered and subjected to principal-component analysis for dimensionality reduction. Integration was applied on a sample (donor) basis.

#### Age group stratification and donor balancing

The dataset was stratified into the following age groups: 19–39, 40–59, 60–69, 70–79 and 80–89. The numbers of male and female donors per age group were balanced using random subsampling.

#### Cluster refinement and T cell subset correction

After integration, an unexpected cluster of T cells was identified, which appeared distinct from the main T cell population and was positioned close to the NK cell cluster. To address this, unsupervised clustering was performed on the normalized and integrated data. The FindNeighbors() function in the Seurat R package was used to construct a shared nearest neighbor graph based on the principal-component analysis reduction, considering the first 15 principal components. Subsequently, clusters were identified using the FindClusters() function with the Louvain algorithm and a resolution of 0.07. The T cell cluster corresponding to the previously observed outlier cells was removed from subsequent analyses.

#### Pseudobulking and data normalization

Pseudobulking was performed using the AggregateExpression() function from the Seurat R package, using donors and cell types as identifiers. The aggregated data were normalized using the NormalizeData() function from Seurat, to remove the impact of different numbers of cells between donors and cell types.

#### Feature selection and sex and cell type effect removal

The dataset was further refined by retaining only genes detected in more than 1% of cells. Data were then scaled using the ScaleData() function. For subsets corresponding to specific cell types, the data were corrected for sex as a potential confounder. For the pooled cell types subset, both sex and cell type were regressed out.

### Comparison of blood-specific and cross-tissue regulatory models

The blood-specific gene regulatory model was trained using a procedure similar to that described above for the cross-tissue regulatory model—that is, a combination of regularized linear regression and stability selection. The expression profiles of pseudobulked left-out cells in the PBMC dataset were reconstructed as described above. Those reconstructed expression profiles were compared (Spearman correlation) to the observed expression profiles in the left-out cells (Extended Data Fig. 1b). We further selected genes with a Spearman correlation difference of 0.2 between the two regulatory models (cross-tissue or blood-specific) as being better predicted by one model over the other. Functional enrichment of these genes was performed using the topGO R package with GO Biological Process terms.

### Gene filtering based on average predictability across age groups

The analysis of age-related changes in predictability was restricted to genes with average predictability across age groups above tissue-specific thresholds. To determine the tissue-specific thresholds, gene–gene relationships in the regulatory model were first randomized. This was achieved by randomly choosing two predictor–target gene pairs and swapping the two targets and repeating this procedure to cover all gene pairs in the regulatory model. The randomized regulatory model is expected to hold no biological significance while maintaining the topological properties of the original model. The randomized model was then used to reconstruct the expression profiles of tissue-specific subsets, including donors of all ages. The Spearman correlation was computed between the reconstructed (predicted) and observed expression patterns of each gene in each tissue. For each tissue, the distribution of Spearman correlation coefficients across all genes was computed, and the right-side tail containing the top 5% values was determined. The value, for each tissue, corresponding to the definition of that 5% tail in the randomized model was then used as threshold for the average predictability of the original model across age groups. Threshold values varied between 0.39 (Thyroid) and 0.76 (Colon – transverse). Genes with average predictability across age groups below this threshold were excluded from the analysis in that tissue.

### Background distribution of predictability slopes

Background distributions of predictability slopes were generated by repeated (100) random permutation of the age vector used as explanatory variable in the linear regression.

### Network propagation of predictability slopes

Network propagation was done using the R package BioNetSmooth (https://github.com/beyergroup/BioNetSmooth) with *α* = 0.2 and a user-defined network. The network defined by our regulatory model was made undirected, by summing the weights of edge pairs between two nodes (edge *i* → *j* and edge *j* → *i*), to capture the full strength of the relationship between gene pairs. The adjacency matrix of the resulting network was then row normalized by dividing each row by the sum of all its entries (each node by its degree). This avoids that highly connected nodes dominate the represented topology^51^.

### GSEA of predictability slopes

GSEA^22^ was performed with the graphical interface of GSEA software version 4.2.3 for Linux in PreRanked mode. For each tissue, genes were ranked according to their age-related predictability slope. The MSigDB^50^ (version 2022.1, updated in August 2022) was used to retrieve hallmark and GO (including Biological Processes, Molecular Functions and Cellular Components) gene sets. Gene sets larger than 100 genes or smaller than 15 genes were excluded from the enrichment analysis.

### Comparison of age-related predictability changes in GTEx and PBMC datasets

PBMCs were pseudobulked by both cell type and donor (as described above) to compute predictability. Expression profiles of each cell type and pooled cell types were reconstructed using the cross-tissue regulatory model. PBMC age-related predictability hits were identified based on the slope *P* value of the linear model Predictability ~ Age (*P* < 0.05).

### Quantification of age-related expression and variance changes

Age-related expression and variance changes were computed after pre-processing of the GTEx data (filtering, normalization and batch correction). For the quantification of expression-level changes with age, we used the R package limma^52^ to fit a linear model with age as explanatory variable, using the function lm.fit() followed by empirical Bayes moderation with the function eBayes(). For the quantification of variance changes with age, we computed the variance within each group and fit a linear model with age as explanatory variable, using R’s base lm() function. In both analyses, age was represented numerically, as the average of the corresponding age group (for example, 25 for the age group 20–29).

### Within-module versus between-module correlations

Hallmark gene sets were downloaded from the MSigDB in March 2023.

### Reporting summary

Further information on research design is available in the Nature Portfolio Reporting Summary linked to this article.

## Supplementary information

Supplementary Tables 1–4

Reporting Summary

## Data Availability

All data were taken from public sources. Source of GTEx data (GTEx version 8): https://www.gtexportal.org/home/downloads/adult-gtex/bulk_tissue_expression PBMC single-cell gene expression data (10.1126/science.abf3041) were downloaded from: https://cellxgene.cziscience.com/collections/dde06e0f-ab3b-46be-96a2-a8082383c4a1 MsigDB hallmark gene sets (version 2022.1) were downloaded from: https://www.gsea-msigdb.org/gsea/msigdb/human/collections.jsp#H The gene co-expression network (10.1371/journal.pcbi.1009849) can be downloaded from: https://github.com/beyergroup/ADImpute/tree/master/data

## Code availability

All code used for this publication can be downloaded from https://github.com/beyergroup/GeneCoordinationLoss.
