# Supplementary material for: Loss of coordination between basic cellular processes in human aging
Source: Nat Aging. 2024 Sep 3;4(10):1432–45. doi: 10.1038/s43587-024-00696-y (PMC11485205; doi:10.1038/s43587-024-00696-y)
Supplement: Supplementary file 1 — Supplementary Tables 1–4 [file 43587_2024_696_MOESM1_ESM.pdf]

nature aging

Supplementary information https://doi.org/10.1038/s43587-024-00696-y

Loss of coordination between basic cellular processes in human aging

In the format provided by the authors and unedited

Table of Contents

Supplementary Table 1……………………… Page 2

Supplementary Table 2……………………… Page 3

Supplementary Table 3……………………… Page 4

Supplementary Table 4……………………… Page 5

Supplementary Table 1: Number of samples per tissue and age group for computation of predictability within age group (Figure 2).

Tissue	20-29	30-39	40-49	50-59	60-69	70-79
Adipose – Visceral	42	42	42	42	42	16
Artery - Tibial	54	54	54	54	54	18
Blood	62	62	62	62	62	21
Brain	46	42	49	49	49	48
Breast – Mammary Tissue	36	36	36	36	36	15
Esophagus - Mucosa	51	51	51	51	51	12
Testis	30	30	30	30	30	-
Thyroid	44	44	44	44	44	20

Supplementary Table 2: Number of samples per tissue and age group, after randomly selecting similar sample numbers per tissue and age group (Figure S6).

Tissue	20-29	30-39	40-49	50-59	60-69	70-79
Adipose - Subcutaneous	30	30	30	30	30	18
Adipose – Visceral	30	30	30	30	30	16
Artery - Tibial	30	30	30	30	30	18
Blood	30	30	30	30	30	21
Brain	30	30	30	30	30	48
Breast – Mammary Tissue	30	30	30	30	30	15
Colon - Transverse	30	30	30	30	30	-
Esophagus - Mucosa	30	30	30	30	30	12
Esophagus - Muscularis	30	30	30	30	30	-
Lung	30	30	30	30	30	17
Muscle - Skeletal	30	30	30	30	30	26
Nerve - Tibial	30	30	30	30	30	18
Skin – Not sun exposed	30	30	30	30	30	22
Skin – Sun exposed	30	30	30	30	30	26
Testis	30	30	30	30	30	-
Thyroid	30	30	30	30	30	20

Supplementary Table 3: Number of genes for which predictability trends with age were computed (Figure 2).

**Tissue**	**# genes**
Adipose – Visceral	4691
Artery - Tibial	3964
Blood	4768
Brain	3728
Breast – Mammary Tissue	4709
Esophagus - Mucosa	5830
Testis	3291
Thyroid	4984

Supplementary Table 4: Number of genes for which predictability trends with age were computed, after randomly selecting similar sample numbers per tissue and age group (Figure S6).

Tissue	# genes
Adipose - Subcutaneous	4569
Adipose – Visceral	4593
Artery – Tibial	4008
Blood	4641
Brain	3708
Breast – Mammary Tissue	4873
Colon – Transverse	2407
Esophagus – Mucosa	5890
Esophagus - Muscularis	4149
Lung	5120
Muscle – Skeletal	2726
Nerve – Tibial	3854
Skin – Not sun exposed	5038
Skin – Sun exposed	5498
Testis	3111
Thyroid	5179
