# Supplementary material for: Loss of coordination between basic cellular processes in human aging
Source: Nat Aging. 2024 Sep 3;4(10):1432–45. doi: 10.1038/s43587-024-00696-y (PMC11485205; doi:10.1038/s43587-024-00696-y)
Supplement: Supplementary file 2 — Reporting Summary [file 43587_2024_696_MOESM2_ESM.pdf]

nature portfolio

Corresponding author(s): Andreas Beyer

Last updated by author(s): Jun 30, 2024

Reporting Summary

Nature Portfolio wishes to improve the reproducibility of the work that we publish. This form provides structure for consistency and transparency in reporting. For further information on Nature Portfolio policies, see our Editorial Policies and the Editorial Policy Checklist.

Statistics

For all statistical analyses, confirm that the following items are present in the figure legend, table legend, main text, or Methods section.

n/a	Confirmed	
☐	☒	The exact sample size (*n*) for each experimental group/condition, given as a discrete number and unit of measurement
☒	☐	A statement on whether measurements were taken from distinct samples or whether the same sample was measured repeatedly
☐	☒	The statistical test(s) used AND whether they are one- or two-sided<br>*Only common tests should be described solely by name; describe more complex techniques in the Methods section.*
☒	☐	A description of all covariates tested
☐	☒	A description of any assumptions or corrections, such as tests of normality and adjustment for multiple comparisons
☐	☒	A full description of the statistical parameters including central tendency (e.g. means) or other basic estimates (e.g. regression coefficient) AND variation (e.g. standard deviation) or associated estimates of uncertainty (e.g. confidence intervals)
☐	☒	For null hypothesis testing, the test statistic (e.g. *F*, *t*, *r*) with confidence intervals, effect sizes, degrees of freedom and *P* value noted<br>*Give P values as exact values whenever suitable.*
☒	☐	For Bayesian analysis, information on the choice of priors and Markov chain Monte Carlo settings
☒	☐	For hierarchical and complex designs, identification of the appropriate level for tests and full reporting of outcomes
☐	☒	Estimates of effect sizes (e.g. Cohen's *d*, Pearson's *r*), indicating how they were calculated

Our web collection on statistics for biologists contains articles on many of the points above.

Software and code

Policy information about availability of computer code

Data collection	No software was used. (No new data was collected.)
Data analysis	Analyses were conducted on R-4.0.3. Plots were created with ggplot2 (v. 3.4.4). R packages used: igraph (v. 1.2.6), org.Hs.eg.db (v. 3.18.0), DESeq2 (v. 1.30.1), seurat (v. 5.0.1), topGO (v. 2.54.0), BioNetSmooth (v. 1.0.0), limma (v. 3.58.1), Further, GSEA v4.2.3 for Linux was used. All code used for this publication can be downloaded from https://github.com/beyergroup/GeneCoordinationLoss.

For manuscripts utilizing custom algorithms or software that are central to the research but not yet described in published literature, software must be made available to editors and reviewers. We strongly encourage code deposition in a community repository (e.g. GitHub). See the Nature Portfolio guidelines for submitting code & software for further information.

Data

Policy information about availability of data

All manuscripts must include a data availability statement. This statement should provide the following information, where applicable:

- Accession codes, unique identifiers, or web links for publicly available datasets
- A description of any restrictions on data availability
- For clinical datasets or third party data, please ensure that the statement adheres to our policy

All data was taken from public sources. Source of GTEx data (GTEx version 8): https://www.gtexportal.org/home/downloads/adult-gtex/bulk_tissue_expression

PBMC single-cell gene expression data (DOI: 10.1126/science.abf3041) was downloaded from:
https://cellxgene.cziscience.com/collections/dde06e0f-ab3b-46be-96a2-a8082383c4a1
MsigDB Hallmark Gene Sets (v2022.1) were downloaded from:
https://www.gsea-msigdb.org/gsea/msigdb/human/collections.jsp#H
The gene co-expression network (DOI: 10.1371/journal.pcbi.1009849) can be downloaded from:
https://github.com/beyergroup/ADImpute/tree/master/data

Research involving human participants, their data, or biological material

Policy information about studies with human participants or human data. See also policy information about sex, gender (identity/presentation), and sexual orientation and race, ethnicity and racism.

Reporting on sex and gender	The GTEx data contains biologically male and female research subjects. We had no influence on the inclusion/exclusion of subjects.
Reporting on race, ethnicity, or other socially relevant groupings	Race, ethnicity, social status or any other grouping of the research subjects was not considered in this study.
Population characteristics	Age of donors was considered, see Methods. Any other covariates were not taken into account.
Recruitment	We had no influence on the recruitment of the research subjects. This was done by the GTEx consortium.
Ethics oversight	See https://www.gtexportal.org. We only used publicly available data that did not require any additional ethics approval.

Note that full information on the approval of the study protocol must also be provided in the manuscript.

Field-specific reporting

Please select the one below that is the best fit for your research. If you are not sure, read the appropriate sections before making your selection.

☒ Life sciences ☐ Behavioural & social sciences ☐ Ecological, evolutionary & environmental sciences

For a reference copy of the document with all sections, see nature.com/documents/nr-reporting-summary-flat.pdf

Life sciences study design

All studies must disclose on these points even when the disclosure is negative.

Sample size	Existing, publicly available data was used. Therefore, we had no influence on the study design or sample numbers. We always aimed to use the maximum number of samples available. For some analyses, samples were randomly down-sampled to have identical numbers of samples per age group. See Methods for more details.
Data exclusions	For some analyses, samples were randomly down-sampled to have identical numbers of samples per age group. See Methods for more details.
Replication	Existing, publicly available data was used. Therefore, we had no influence on the study design or sample numbers. We had no influence on replication. We did not exclude replicates based on quality criteria. For some analyses, samples were randomly down-sampled to have identical numbers of samples per age group. See Methods for more details. Conclusions were robust to different sub-samples of the data, i.e. independent of specific replicates.
Randomization	Since this is not a case-control study no randomization was necessary.
Blinding	Since this is not a case-control study, no blinding was necessary.

Reporting for specific materials, systems and methods

We require information from authors about some types of materials, experimental systems and methods used in many studies. Here, indicate whether each material, system or method listed is relevant to your study. If you are not sure if a list item applies to your research, read the appropriate section before selecting a response.

Materials & experimental systems

n/a	Involved in the study
☒	☐ Antibodies
☒	☐ Eukaryotic cell lines
☒	☐ Palaeontology and archaeology
☒	☐ Animals and other organisms
☒	☐ Clinical data
☒	☐ Dual use research of concern
☒	☐ Plants

Methods

n/a	Involved in the study
☒	☐ ChIP-seq
☒	☐ Flow cytometry
☒	☐ MRI-based neuroimaging
